# Evaluating the Renal Implications of Intensive Versus Standard Blood Pressure Control in Chronic Kidney Disease: A Systematic Review of Randomized Trials

**DOI:** 10.7759/cureus.91323

**Published:** 2025-08-31

**Authors:** Kirshan Lal, Ahmad Mohammad, Muhammad Umar Tariq, Nimra Jabeen, Sumbal Shaikh, Shivam Singla, Sara Zubair Ahmed, Ramesh Pant, Ahmed Khan, Muhammad A Basit, Aqib Imran

**Affiliations:** 1 Pediatrics, Shaheed Mohtarma Benazir Bhutto Medical University, Larkana, PAK; 2 Internal Medicine, Hurley Medical Center, Flint, USA; 3 Internal Medicine, King Edward Medical University, Lahore, PAK; 4 Internal Medicine, HITEC Institute of Medical Sciences, Taxila, PAK; 5 Internal Medicine, Chandka Medical College, Larkana, PAK; 6 Internal Medicine, TidalHealth Penninsula Regional, Salisbury, USA; 7 Internal Medicine, Baqai Medical University, Karachi, PAK; 8 Internal Medicine, Maya Metro Hospital, Dhangadhi, NPL; 9 Internal Medicine, Liaquat College of Medicine & Dentistry, Karachi, PAK; 10 Internal Medicine, Quaid-e-Azam Medical College, Bahawalpur, PAK; 11 Internal Medicine, Nishtar Medical University, Multan, PAK

**Keywords:** albuminuria, blood pressure targets, chronic kidney disease, egfr, intensive bp control, proteinuria, randomized controlled trials, renal outcomes

## Abstract

This systematic review evaluates the renal implications of intensive versus standard blood pressure (BP) control in patients with chronic kidney disease (CKD). PubMed, Scopus, and Embase were searched through June 2025 for randomized controlled trials (RCTs) and high-quality post hoc analyses comparing intensive (systolic BP <120 mmHg) versus standard targets (<140 mmHg) in adults with CKD. Four studies involving 20,803 participants with a median follow-up of 2 to 3.2 years were included. Intensive BP control was associated with a higher incidence of ≥40% estimated glomerular filtration rate (eGFR) decline in non-albuminuric patients (hazard ratio up to ~4.5), but showed a significant reduction in proteinuria among patients with diabetic nephropathy (mean reduction ~1,200 mg/24h). Across all studies, the cardiovascular benefits of intensive BP lowering were consistent regardless of baseline kidney function. Overall, intensive BP control does not cause long-term renal harm, though early eGFR decline may reflect hemodynamic rather than structural changes. Patients with albuminuria appear to gain the most renal benefit, supporting individualized BP targets. Clinically, tailoring BP goals to albuminuria status may optimize renal outcomes while maintaining cardiovascular protection.

## Introduction and background

Chronic kidney disease (CKD) affects over 10% of the global population and represents a significant contributor to morbidity, mortality, and healthcare burden [1]. One of the most critical modifiable risk factors in CKD progression is hypertension, which not only accelerates the decline of renal function but also substantially increases the risk of cardiovascular events [2]. In patients with CKD, elevated blood pressure is often both a cause and a consequence of impaired renal function, forming a vicious cycle that can lead to end-stage kidney disease (ESKD) if not properly managed [3].

Blood pressure management in CKD has traditionally aimed for conservative targets; however, over the past two decades, there has been increasing interest in whether intensive blood pressure control, typically targeting a systolic BP of <120 mmHg, offers superior renal protection compared to standard targets (e.g., <140 mmHg) [4]. Large-scale trials have demonstrated cardiovascular benefits with intensive control in high-risk patients, yet the renal outcomes remain nuanced. While some studies suggest potential slowing of estimated glomerular filtration rate (eGFR) decline and reductions in albuminuria (a surrogate marker of glomerular injury), others raise concerns about acute GFR drops or adverse renal effects, especially in subgroups like the elderly or those with diabetic nephropathy [5].

In this context, albuminuria and glomerular filtration rate (GFR) remain central clinical markers for assessing kidney disease progression and therapeutic response [6]. While several landmark trials have explored the effects of intensive blood pressure (BP) lowering, their renal outcomes have been inconsistent, some suggesting benefits in proteinuria reduction, others showing accelerated eGFR decline, particularly in non-albuminuric patients [7]. Moreover, most existing reviews have prioritized cardiovascular endpoints, leaving uncertainty about the renal safety and subgroup-specific implications of intensive BP control. This gap underscores the need for a focused synthesis of evidence addressing kidney-related outcomes. Therefore, this systematic review evaluates whether intensive BP control (target systolic <120 mmHg), compared with standard targets (<140 mmHg), influences renal function decline (eGFR change) and albuminuria in adults with CKD, based on data from randomized controlled trials and high-quality post hoc analyses.

## Review

Materials and methods

Study Design and Protocol Framework

This systematic review was conducted following the Preferred Reporting Items for Systematic Reviews and Meta-Analyses (PRISMA) guidelines to ensure methodological rigor and transparency [8]. The review was conceptualized using the PICO (Population, Intervention, Comparison, Outcome) framework [9]: the Population included adults with chronic kidney disease (CKD); the Intervention was intensive blood pressure (BP) control; the Comparison was standard BP control; and the Outcomes assessed were changes in eGFR, albuminuria or proteinuria levels, and clinical implications related to renal progression. The study protocol was developed in accordance with PRISMA criteria but was not prospectively registered. The lack of prospective registration (e.g., PROSPERO) may introduce reporting bias and should be considered when interpreting results.

Eligibility Criteria

Eligible studies were randomized controlled trials (RCTs) or post hoc analyses of RCTs that compared intensive versus standard BP targets in patients with CKD. Studies were included if they reported on at least one renal outcome such as eGFR change, albuminuria or proteinuria progression, or renal adverse events. Articles were limited to English-language publications involving human subjects and were required to have clearly defined BP targets. Exclusion of non-English studies could introduce language bias, which should be acknowledged. Observational studies were excluded to maintain a focus on randomized evidence and minimize confounding, although this may have omitted longer-term data on renal outcomes that are sometimes reported in observational cohorts. Editorials, conference abstracts, animal studies, and reviews were also excluded.

Search Strategy and Data Sources

A comprehensive literature search was conducted across three major databases: PubMed, Scopus, and Embase. The search covered publications from database inception through June 2025. Search terms included combinations of Medical Subject Headings (MeSH) and free-text keywords such as “chronic kidney disease,” “blood pressure control,” “eGFR,” “albuminuria,” “proteinuria,” “SPRINT,” “intensive blood pressure,” and “renal outcomes.” Boolean operators were used to enhance search sensitivity and specificity. Reference lists of the included articles and relevant reviews were also manually screened to identify additional eligible studies.

Study Selection and Screening

All identified records were imported into reference management software (EndNote X9, Clarivate, London, UK), and duplicates were removed. Screening was performed using Rayyan (Rayyan Systems Inc., Cambridge, MA, USA) software to facilitate blinded, independent assessment of abstracts and full texts. Two independent reviewers screened titles and abstracts for relevance, followed by full-text assessments to confirm eligibility. Discrepancies were resolved through discussion and consensus, with arbitration by a third reviewer if needed. A PRISMA flow diagram was constructed to illustrate the selection process and reasons for study exclusion at each stage.

Data Extraction

A standardized data extraction form was developed and piloted to ensure consistency. Extraction was performed independently by two reviewers to reduce errors and bias. Extracted variables included study design, sample size, population characteristics, CKD definition and stage, BP targets in both intervention and control arms, follow-up duration, renal outcomes (eGFR changes, albuminuria or proteinuria), and key findings. Where necessary, corresponding authors were contacted to clarify ambiguous or missing data.

Risk of Bias Assessment

Each included study underwent a formal risk of bias evaluation using the Cochrane Risk of Bias 2.0 tool for randomized trials [10]. Domains assessed included randomization process, deviations from intended interventions, missing outcome data, measurement of the outcome, and selection of reported results. Studies were classified as low risk, some concerns, or high risk based on this tool. The assessments were performed independently by two reviewers and confirmed by consensus. A summary table and figure showing the risk of bias across included studies are provided in the Results section.

Data Synthesis

Given the heterogeneity in study design, outcome reporting, baseline kidney function, and definitions of albuminuria, quantitative pooling (meta-analysis) was not feasible. Variations in follow-up duration, endpoints (e.g., absolute vs. percentage eGFR decline), and measurement techniques would have limited the validity of pooled estimates. Therefore, a narrative synthesis was undertaken. Key trends were identified and compared across studies, with special attention to the influence of baseline albuminuria, stage of CKD, and duration of follow-up.

Results

Study Selection Process

**Figure 1:**
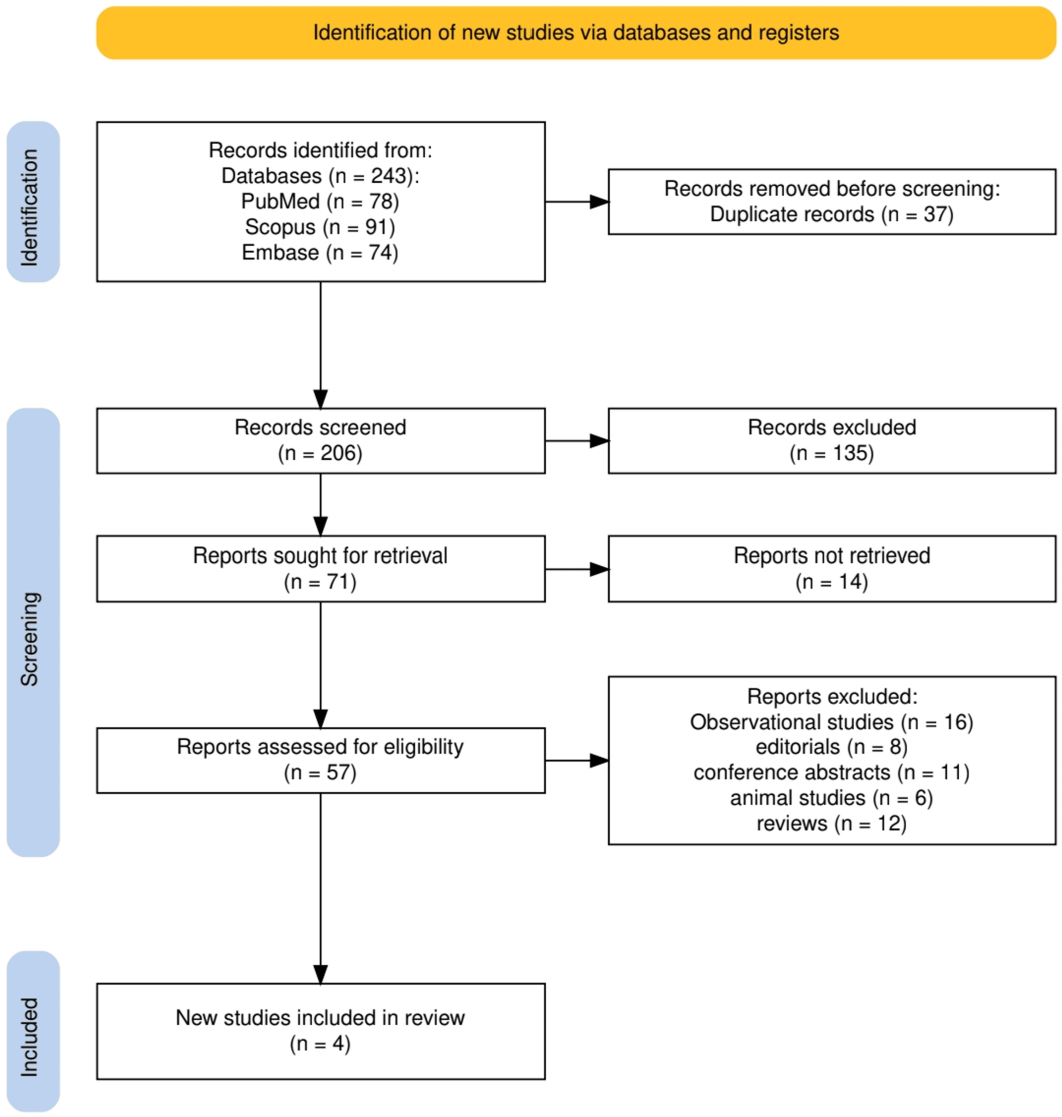
PRISMA flow diagram showing the study selection process. PRISMA, Preferred Reporting Items for Systematic Reviews and Meta-Analyses.

The study selection process is detailed in Figure 1, which illustrates the systematic approach undertaken in accordance with PRISMA 2020 guidelines. An initial total of 243 records were identified through comprehensive searches of PubMed (78), Scopus (91), and Embase (74). After the removal of 37 duplicates, 206 records remained for title and abstract screening. Of these, 135 were excluded for not meeting eligibility criteria, and 71 full-text articles were assessed. Fourteen could not be retrieved, and 57 full-text reports were evaluated for eligibility. Ultimately, 53 studies were excluded based on predefined criteria, including observational design, editorials, conference abstracts, animal studies, and review articles. This process resulted in four studies being included in the final systematic review.

Characteristics of the Selected Studies

Table 1 summarizes the key characteristics and findings of the four studies included in this systematic review. All studies were based on randomized controlled trial designs or post hoc analyses of major RCTs and evaluated the impact of intensive versus standard blood pressure targets in patients with varying degrees of chronic kidney disease. Follow-up durations ranged around 2 to 3.2 years, with diverse definitions of CKD based on eGFR or albuminuria. While some studies highlighted the protective role of intensive BP control on cardiovascular outcomes, findings regarding renal outcomes, such as eGFR decline and proteinuria progression, were mixed and often modified by baseline renal function or biomarker profiles.

**Table 1:** Summary of randomized controlled trials and post hoc analyses comparing intensive versus standard blood pressure targets in chronic kidney disease. ACR, albumin-to-creatinine ratio; ACEI, angiotensin-converting enzyme inhibitor; BP, blood pressure; CKD, chronic kidney disease; CrCl, creatinine clearance; CV, cardiovascular; eGFR, estimated glomerular filtration rate; HF, heart failure; HR, hazard ratio; MAP, mean arterial pressure; RCT, randomized controlled trial; SBP, systolic blood pressure; SPRINT, Systolic Blood Pressure Intervention Trial; UACR, urine albumin-to-creatinine ratio; β2m, beta-2 microglobulin.

Study (author, year)	Study design	Population (sample size)	CKD definition/stage	BP targets (intensive vs. standard)	Follow-up duration	eGFR outcome	Albuminuria/proteinuria outcome	Key findings
Jotwani et al., 2020 [11]	Randomized controlled trial (SPRINT Substudy)	2,428 participants with eGFR <60 mL/min/1.73 m²	CKD defined by eGFR <60 mL/min/1.73 m² (mean 46 ± 11)	Intensive: SBP <120 mmHg; Standard: SBP <140 mmHg	Median follow-up not directly stated; inferred ~3 years from SPRINT	eGFR declined more rapidly in participants with higher tubular injury biomarkers (e.g., β2m); this association was attenuated in the intensive BP group	Albuminuria included in models, but not a primary outcome; baseline ACR used as adjustment variable	Tubular biomarkers (uromodulin, β2m) predicted eGFR decline; effect was less pronounced in intensive BP group
Chang et al., 2020 [12]	Post hoc analysis of RCT (SPRINT)	8,885 non-diabetic adults ≥50 years with high CV risk (1,723 with albuminuria; 7,162 without)	Not explicitly staged; subgroup analysis by baseline albuminuria (ACR ≥30 mg/g)	Intensive: SBP <120 mmHg; Standard: SBP <140 mmHg	Median 3.1 years	≥40% eGFR decline occurred more frequently with intensive BP control, especially in participants without albuminuria (HR 4.55 vs. 1.48); absolute differences were small	Baseline albuminuria (ACR ≥30 mg/g) used as stratifier; incidence rates of decline higher in albuminuric group	Albuminuria modified the relative but not absolute effect of intensive BP control on eGFR decline; did not alter CV or mortality benefit
Lewis et al., 1999 [13]	Randomized controlled trial (ACEI + BP target)	129 patients with type 1 diabetes and nephropathy (creatinine <4.0 mg/dL)	Diabetic nephropathy with preserved renal function (iothalamate clearance ~62–64 mL/min/1.73 m² at baseline)	Group I: MAP ≤92 mmHg (intensive); Group II: MAP 100–107 mmHg (standard)	2 years minimum	No statistically significant difference in GFR decline (measured via iothalamate clearance and CrCl) between groups	Significant reduction in 24-hour proteinuria in intensive group (535 mg vs. 1,723 mg; p = 0.02)	Intensive BP control + ACEI reduced proteinuria, though not GFR decline; supports use of intensive MAP goals for proteinuria control in diabetic nephropathy
Vaduganathan et al., 2021 [14]	Post hoc analysis of RCT (SPRINT substudy)	9,361 participants ≥50 years with high CV risk (2,650 with eGFR <60 mL/min/1.73 m²; 248 with UACR >300 mg/g)	Stratified by eGFR and UACR levels; CKD defined as eGFR <60 or UACR >30	Intensive: SBP <120 mmHg; Standard: SBP <140 mmHg	Median 3.2 years (range 0–4.8 years)	eGFR <60 associated with higher HF and CV death risk; BP treatment effect consistent across eGFR strata	Albuminuria (UACR >300 mg/g) associated with higher HF risk; did not modify BP treatment effect	Intensive BP control reduced HF risk consistently; neither eGFR nor UACR significantly modified this effect; both were strong predictors of HF/CV outcomes

Risk of Bias Assessment

Table 2 presents the risk of bias assessment for the included studies using standard domains, including randomization, deviations from intended interventions, outcome data, and reporting transparency. Three of the studies demonstrated a consistently low risk of bias across all domains, reflecting strong methodological rigor and adherence to protocol-defined outcomes. One study, although based on the SPRINT (Systolic Blood Pressure Intervention Trial) framework, raised some concerns due to its exploratory nature and outcomes not being pre-specified in the original protocol. Overall, the methodological quality of the included studies supports the reliability of the findings presented in this review.

**Table 2:** Risk of bias assessment of included studies using the Cochrane RoB 2.0 tool. ACR, albumin-to-creatinine ratio; BP, blood pressure; CrCl, creatinine clearance; eGFR, estimated glomerular filtration rate; HF, heart failure; RCT, randomized controlled trial; RoB 2.0, Cochrane risk of bias tool (version 2.0); SPRINT, Systolic Blood Pressure Intervention Trial.

Study (author, year)	Randomization process	Deviations from intended interventions	Missing outcome data	Measurement of outcome	Selective reporting	Overall risk of bias	Comments
Jotwani et al., 2020 [11]	Low risk (SPRINT randomization maintained)	Low risk (intended interventions preserved)	Low risk (complete follow-up reported)	Low risk (objective eGFR measures)	Low risk	Low risk	Biomarker-based analysis nested within SPRINT; robust and well-reported
Chang et al., 2020 [12]	Low risk (SPRINT RCT population)	Low risk (predefined BP targets, ACR used for stratification)	Low risk	Low risk	Low risk (results transparently reported)	Low risk	Although post hoc, the analysis was protocol-aligned and statistically sound
Lewis et al., 1999 [13]	Low risk (proper RCT design)	Low risk (clear BP targets and intervention adherence)	Low risk (follow-up period completed)	Low risk (iothalamate clearance, CrCl used)	Low risk	Low risk	Classical trial with predefined endpoints; no indication of reporting bias
Vaduganathan et al., 2021 [14]	Low risk (SPRINT RCT backbone)	Some concerns (secondary, exploratory HF outcomes)	Low risk	Low risk	Some concerns (not pre-specified in original SPRINT)	Some concerns	Excellent quality but secondary analysis of outcomes not primary in SPRINT protocol

Discussion

This systematic review evaluated the impact of intensive versus standard blood pressure (BP) control on renal function decline and albuminuria in patients with chronic kidney disease (CKD), synthesizing data from four randomized controlled trials [11-14] including substudies from SPRINT and diabetic nephropathy cohorts. Across the reviewed studies, intensive BP control, typically targeting systolic BP <120 mmHg, did not consistently demonstrate superior outcomes for glomerular filtration rate (GFR) preservation compared to standard BP targets (e.g., <140 mmHg). In Chang et al., a ≥40% decline in eGFR was more frequent in the intensive group (hazard ratio (HR) 4.55 in non-albuminuric vs. 1.48 in albuminuric patients), though absolute differences were small [12]. In contrast, Jotwani et al. found that urinary biomarkers such as β2-microglobulin were predictive of eGFR decline, but this association was attenuated with intensive BP control, suggesting a potential protective renal effect [11]. While Lewis et al. observed no significant difference in GFR decline between groups, they did report a significant reduction in proteinuria (535 mg vs. 1,723 mg/24 h; p = 0.02) in the intensive group [13]. Similarly, Vaduganathan et al. showed that intensive BP lowering reduced heart failure (HF) risk across eGFR and albuminuria strata, but renal function was not significantly preserved [14]. Overall, these findings indicate that intensive BP control may reduce albuminuria and proteinuria progression in albuminuric and diabetic CKD populations, while its effects on eGFR decline are more variable-showing potential protective impact in biomarker-defined high-risk patients but a higher risk of early functional decline in non-albuminuric individuals.

The heterogeneity observed in renal outcomes across these trials likely reflects the complex renal hemodynamic responses to BP reduction, particularly in CKD subtypes. Intensive BP lowering can transiently reduce eGFR due to decreased intraglomerular pressure, a phenomenon commonly seen with ACE inhibitors or aggressive diuresis [15]. In patients without baseline albuminuria, such as those highlighted in Chang et al., this hemodynamic drop in GFR may be more pronounced, possibly explaining the elevated HR (4.55) for eGFR decline in the intensive arm [12]. Conversely, in albuminuric patients, where glomerular hyperfiltration and capillary wall stress are major contributors to progression, lowering BP may have more durable structural benefits, as suggested by the reduced proteinuria observed in Jotwani et al. and Lewis et al. [11,13]. Tubular markers in Jotwani et al. further support this notion, showing that early tubular injury correlates with GFR loss, and that this risk may be mitigated under tighter BP control [11]. Importantly, these biomarkers hold potential clinical value as early warning tools to identify patients most likely to benefit from intensive BP control while avoiding overtreatment in lower-risk individuals. Their integration into routine practice could enhance risk stratification and guide personalized BP target selection in CKD management. Thus, while eGFR changes may not always reflect irreversible damage, reductions in albuminuria and injury biomarkers under intensive BP targets imply renoprotective benefits in selected patients, particularly those with baseline signs of glomerular stress.

The findings of our systematic review are largely consistent with landmark trials such as SPRINT [12], which demonstrated that targeting a systolic blood pressure (SBP) below 120 mmHg led to significant cardiovascular benefits but also caused an acute decline in eGFR. Notably, this decline did not necessarily translate into long-term kidney damage in non-albuminuric patients. Similarly, the AASK (African-American Study of Kidney Disease and Hypertension) trial supported intensive BP control among African American patients with hypertensive CKD [16,17], showing slower progression of kidney disease without increased adverse renal events. In contrast, the ACCORD trial did not show renal benefits in diabetic patients, suggesting that the impact of BP lowering may differ by underlying disease mechanism [18,19]. Our review confirms the emerging view that albuminuria status, rather than eGFR alone, should guide BP targets. These conclusions align with but also refine current KDIGO (Kidney Disease: Improving Global Outcomes) and ACC/AHA (American College of Cardiology/American Heart Association) guidelines [20,21], which broadly endorse lower BP targets for CKD but do not yet fully incorporate stratification by proteinuria levels.

The collective evidence indicates that a uniform BP target may not be appropriate for all CKD patients. Individualizing BP goals, especially by considering albuminuria or tubular injury markers, could prevent overtreatment in low-risk populations while ensuring adequate control in those most vulnerable to progression [22]. Patients with low eGFR but minimal or no albuminuria may experience hemodynamic decreases in GFR with intensive BP treatment that are not indicative of true nephron loss. In contrast, those with elevated albuminuria are more likely to benefit from aggressive BP control, as it slows progression and reduces cardiovascular risk. Clinicians should consider a more nuanced approach, incorporating not only eGFR and albuminuria but also age, comorbidities, and possibly tubular biomarkers, to guide decision-making in BP management for CKD.

One of the key insights from this review is that an early decline in eGFR with intensive BP lowering may represent a functional, hemodynamically mediated change rather than true progression of CKD, particularly in patients without albuminuria. This reframing challenges the traditional interpretation of creatinine-based changes as a surrogate for irreversible damage and suggests that nephrologists should be cautious in overreacting to modest eGFR declines in the absence of proteinuria [23]. Furthermore, our synthesis highlights albuminuria not just as a marker of risk, but as a potential effect modifier that influences how the kidneys respond to antihypertensive therapy. Integrating emerging tubular biomarkers (such as NGAL, KIM-1, or uromodulin) into practice may further refine patient selection and optimize BP targets in the future [24,25].

This review offers several strengths, including its focus on randomized controlled trials, which provide the highest level of clinical evidence, and its attention to renal outcomes such as eGFR decline and albuminuria progression. The use of consistent BP thresholds across most trials improves the validity of our comparisons. Additionally, by including both diabetic and non-diabetic CKD populations, the findings have wide applicability. However, the review is not without limitations. Significant heterogeneity existed across studies in terms of participant demographics, baseline kidney function, and definitions of albuminuria. Most trials had a follow-up period of less than five years, limiting the ability to assess long-term renal preservation. The post hoc nature of some renal outcome analyses also introduces the risk of selection and reporting bias.

Future research should prioritize large, prospective RCTs specifically designed to evaluate the renal effects of intensive BP control across diverse CKD subgroups. There is a pressing need to determine whether early eGFR declines with treatment are reversible and whether they translate into long-term benefit or harm. Additionally, clinical trials incorporating biomarkers of tubular injury or inflammation could help identify patients who are most likely to benefit (or be harmed) by aggressive BP lowering [26,27]. Finally, studies should explore the role of personalized BP targets guided by both traditional (eGFR, albuminuria) and novel (tubular) markers to more precisely tailor therapy and improve outcomes for patients with CKD.

## Conclusions

This systematic review underscores the nuanced relationship between intensive blood pressure control and renal outcomes in patients with chronic kidney disease. While aggressive BP lowering may initially lead to a decline in eGFR, particularly in non-albuminuric patients, this change is often hemodynamic rather than a marker of true kidney injury. At the same time, patients with albuminuria appear to derive the most benefit, supporting the integration of proteinuria assessment into BP target decision-making. These findings advocate for a more individualized approach to blood pressure management in CKD, moving beyond one-size-fits-all targets toward strategies informed by both functional and structural kidney markers. Future research should further explore this tailored approach by incorporating emerging biomarkers and evaluating long-term outcomes. Ultimately, optimizing BP targets based on patient phenotype could improve renal preservation while minimizing harm, enhancing the precision of nephrology care.
